# Advancement of Peptide Nanobiotechnology via Emerging Microfluidic Technology

**DOI:** 10.3390/mi10100627

**Published:** 2019-09-20

**Authors:** Kiat Hwa Chan, Jonathan Jen Jie Tay

**Affiliations:** 1 Division of Science, Yale-NUS College, 16 College Avenue West, Singapore 138527, Singapore; 2 Department of Chemistry, National University of Singapore, 3 Science Drive 3, Singapore 117543, Singapore; e0020840@u.nus.edu

**Keywords:** self-assembly, peptides, nanoparticles, low dispersity, microgels, miniaturized cell cultures, microfluidics

## Abstract

Peptide nanotechnology has experienced a long and enduring development since its inception. Many different applications have been conceptualized, which depends on the functional groups present on the peptide and the physical shape/size of the peptide nanostructures. One of the most prominent nanostructures formed by peptides are nanoparticles. Until recently, however, it has been challenging to engineer peptide nanoparticles with low dispersity. An emerging and promising technique involves the utility of microfluidics to produce a solution of peptide nanoparticles with narrow dispersity. In this process, two or more streams of liquid are focused together to create conditions that are conducive towards the formation of narrowly dispersed samples of peptide nanoparticles. This makes it possible to harness peptide nanoparticles for the myriad of applications that are dependent on nanoparticle size and uniformity. In this focus review, we aim to show how microfluidics may be utilized to (1) study peptide self-assembly, which is critical to controlling nanostructure shape and size, and peptide-interface interactions, and (2) generate self-assembling peptide-based microgels for miniaturized cell cultures. These examples will illustrate how the emerging microfluidic approach promises to revolutionize the production and application of peptide nanoparticles in ever more diverse fields than before.

## 1. Introduction

Peptides are ubiquitous in nature. They fulfill wide-ranging roles in including but not limited to, cell-cell recognition (epitopes) [1], metabolism (enzymes) [2], and cellular structure (actin) [3] in living systems. Being such a common motif in living systems, peptides are generally biocompatible and non-immunogenic and thus have been harnessed in biomaterials for various purposes [4,5,6]. A particularly attractive feature of peptide-based biomaterials compared to other common biomaterials is that they may be slowly degraded over time after they have fulfilled their intended functions. This is extremely advantageous as this allows the living system to potentially return to its original state before disease. For instance, in the case of traumatic bone injuries, a course of treatment may involve metallic implants. While it is possible to modify the surface of the metallic implant to reduce rejection by the body [7], it will be better if a suitable biomaterial can be employed to promote bone regeneration and subsequently degrade to leave behind only the new bone tissue [8]. In fact, peptides are so versatile that they have been exploited in a far wider range of applications other than tissue engineering. They include, but are not limited to, drug delivery [9,10,11], carbon monoxide delivery for modulating physiological responses [12,13], and even crude oil recovery from the ocean [14,15,16].

In the examples cited above, the peptides were utilized in the form of gels, a semi-solid state in which the peptide is typically present as only a small proportion of the dry mass of the material; the remaining mass of the gel is largely made up of the solvent. For hydrogels in biological applications, the solvent is water whereas for organogels in the case of crude oil recovery, the solvent would be an organic liquid. In particular for hydrogels, the voluminous space within the hydrogel is able to accommodate a large amount of nutrients that are required to support cellular proliferation, thus making hydrogels an outstanding material for tissue engineering applications [17,18]. Another biological application that capitalizes on the abundant space within hydrogels is drug delivery, which permits maximization of drug encapsulation within the hydrogel. At the site of hydrogel application, the encapsulated drug would be released in a controlled manner in order to achieve the desired drug release profile [19,20,21].

**Figure 1:**
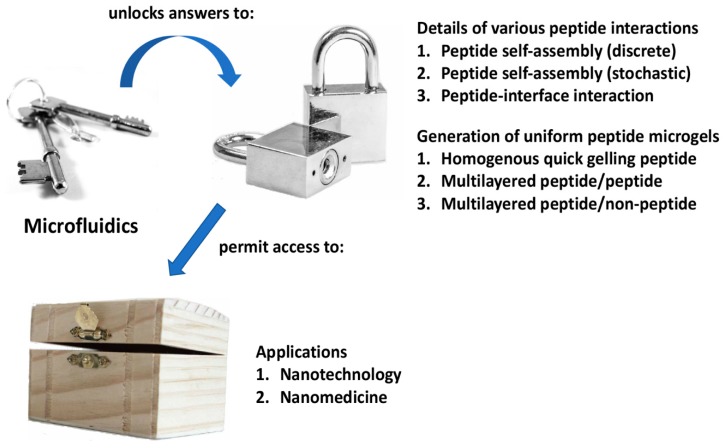
Summary of how microfluidics can be a key to unlock the answers to critical questions that will permit access to the nanobiotechnological potential of self-assembling peptides.

Peptide hydrogels, however, are not the only materials that can facilitate drug delivery; self-assembling peptide nanoparticles are also a potentially promising route [5]. By decorating a nanoparticle with suitable epitopes, it may be possible to transport an encapsulated drug within the nanoparticle specifically to the target site of action [22,23,24]. This is a critical advantage that is not readily achieved with peptide hydrogels. To date, however, reports that discuss the utility of peptide nanoparticles for drug delivery are fairly few [5,25]. In most cases, the peptides are conjugated with established nanoparticle platforms [26], instead of being the primary constituent of the nanoparticle. A potential drawback of peptide nanoparticles that may limit the development of self-assembling peptide-based nanoparticles for drug delivery is that they may be degraded by proteases within the body, although this may be circumvented by utilizing unnatural D-amino acid-based peptide nanoparticles. Another drawback is the control of the size distribution of nanoparticles, which is vitally important to fulfilling its functions [27]. Yet, this is precisely the property that is generally difficult to control for [28]. With the suitable design of the peptide, it is certainly possible to attain a sample of peptide nanoparticles with narrow dispersity [29]. For example, a 20-amino acids peptide has been found to be able to form peptide nanoparticles with an average diameter of 731 nm and a dispersity of only 0.09 that are capable of delivering a pro-apoptosis peptide into cells [30]. This is a very specialised and long peptide sequence. For more facile and widespread adaptation to different drug payloads, it would be ideal to utilize shorter sequences. However, the self-assembly of short peptides into particles is likely to lead to a disperse distribution [31],

A method to control peptide self-assembly that is starting to gain traction with researchers is microfluidics [32]. This is a technique in which microchannels are utilized to control precisely the volume/quantity of substances flowing past a certain point. By adjusting critical parameters, it becomes possible to achieve a low dispersity or uniform sample of nanoparticles [33]. Firstly, the width of the microchannels serves as a physical limit to the quantity of substances that may be mixed together per unit time. Secondly, the speed at which different microstreams of liquids are focused together leads to different mixing times, which in turn leads to different particle sizes. Thirdly, the angle at which the various streams of liquids are focused together also impacts on the eventual size of the particles formed. These parameters have been successfully manipulated to control the self-assembly of nanoparticles involving polysulfone [34], surfactant-polyelectrolyte [35], DNA [36,37,38], among many other systems [39,40,41], with impressive results. To date, however, there are only a limited number of reports that pertain to the utility of microfluidics to control peptide nanoparticle formation, reflecting the incipient development of this field. Two areas stand out: (1) the study of peptide self-assembly and (2) the development of self-assembling peptide-based microenvironments for miniaturized cell cultures. Thus, this focus review aims to provide a comprehensive coverage of how microfluidics may be utilized to advance peptide nanotechnology (Figure 1). Given the impressive results that have been achieved thus far, further exciting developments should certainly be anticipated.

### 2.1. Utility of Microfluidics to Study Peptide Self-Assembly and Peptide-Interface Interaction

In order to fully harness the potential of peptide self-assembly for nanotechnology, it is crucial to understand the factors that affect the very process of self-assembly so that any desired nanostructure may be constructed from a bottom-up approach with absolute precision. It is with the related intention to study the formation of amyloids, aggregated proteins that have been implicated in neurodegenerative diseases such as Alzheimer’s disease and Parkinson’s disease, that microfluidics has been employed to understand this detrimental process [42,43] so that therapeutic approaches may be refined to tackle it. While the chemical motifs that encourage/discourage peptide self-assembly have been studied and are known to a certain extent [44], the physical factors are largely unknown. Naturally, it is crucial that this gap of understanding is filled. As with other self-assembly systems [45], microfluidics has been utilized to study peptide self-assembly.

**Figure 2:**
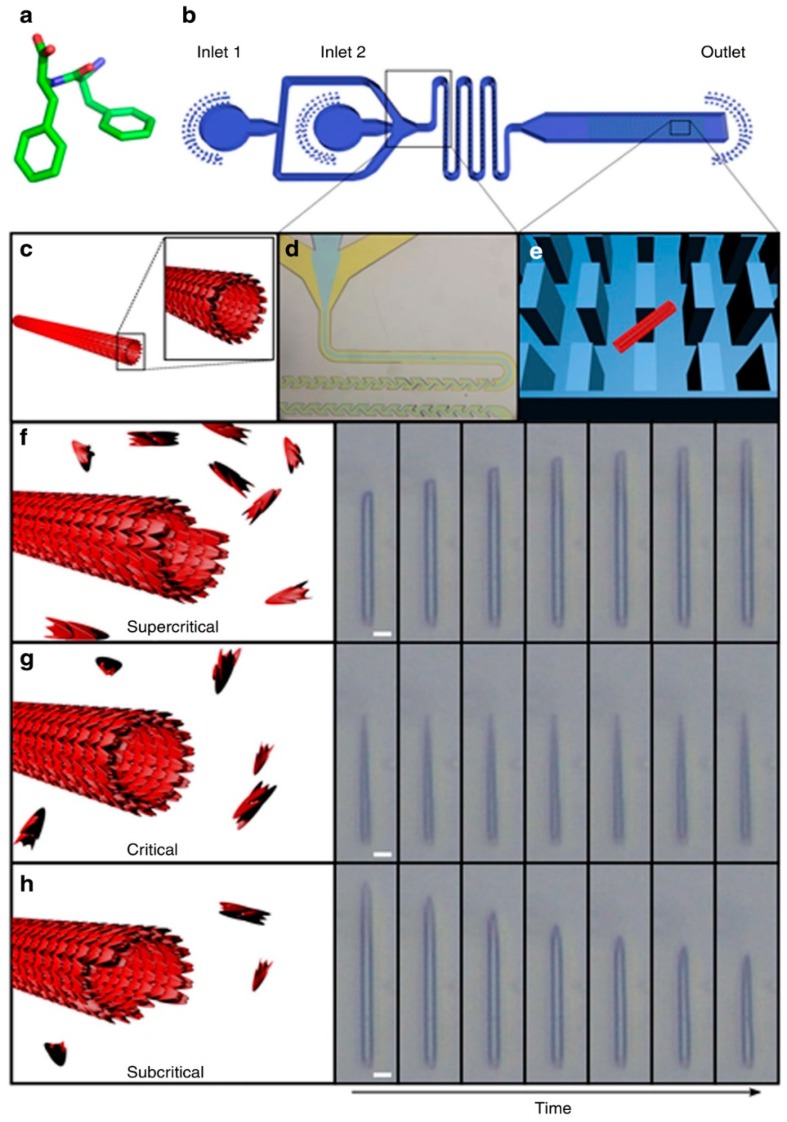
Studying the self-assembly of Phe-Phe at different concentrations of Phe-Phe in the microfluidic flow. (**a**) Molecular structure of Phe-Phe, (**b**) schematic of the microfluidic set-up for the study of Phe-Phe self-assembly, (**c**) illustration of the cross-section of a Phe-Phe nanotube, (**d**) expansion of the microfluidic section where microdroplets are formed, (**e**) illustration of how a Phe-Phe nanotube (red rectangle) can be trapped among the micro-pillars (blue rectangles) within the microfluidic chip, (**f**–**h**) illustration and light microscopic visualization of nanotube lengthening, unchanging, and shortening under flows of supercritical, critical, and subcritical concentrations of Phe-Phe respectively. Reprinted by permission from Macmillan Publishers Ltd.: Nature Communications, Arnon, Z. A. et al. Dynamic microfluidic control of supramolecular peptide self-assembly. Nat. Commun. **2016**, *7*, 13190. doi: 10.1038/ncomms13190, copyright 2016.

In order to achieve this, Arnon et al. employed a microchip with micro-scale pillars to trap nanotubes of diphenylalanine (Phe-Phe; Figure 2a) and cyclo-Phe-Phe (a cyclic form of Phe-Phe) [46,47]. With the nanotubes trapped among the micro-pillars within the chip, the concentrations of peptide monomers may be varied. Due to the small volumes involved, it is possible to adjust quickly and precisely the local concentration of peptide monomer (around the nanotubes) to study the kinetics of self-assembly. As Figure 2 illustrates, the assembly/disassembly (i.e., growth) of the nanotube depended on whether the concentration of the peptide is subcritical, critical, or supercritical. Although this outcome may be expected, e.g., flowing subcritical concentrations of monomer past the nanotube would lead to the progressive dissociation of monomer from the nanotube, shortening it, what is unexpected is the directionality of growth of the nanotubes. As light microscopy reveals, the growth of Phe-Phe is only unidirectional (Figure 2f–h). On the other hand, the growth of cyclo-Phe-Phe is bidirectional, as can be expected. Such a phenomenon has not been observed before and should be (and can be) related to the relative arrangement of the chemical motifs on the molecular level.

**Figure 3:**
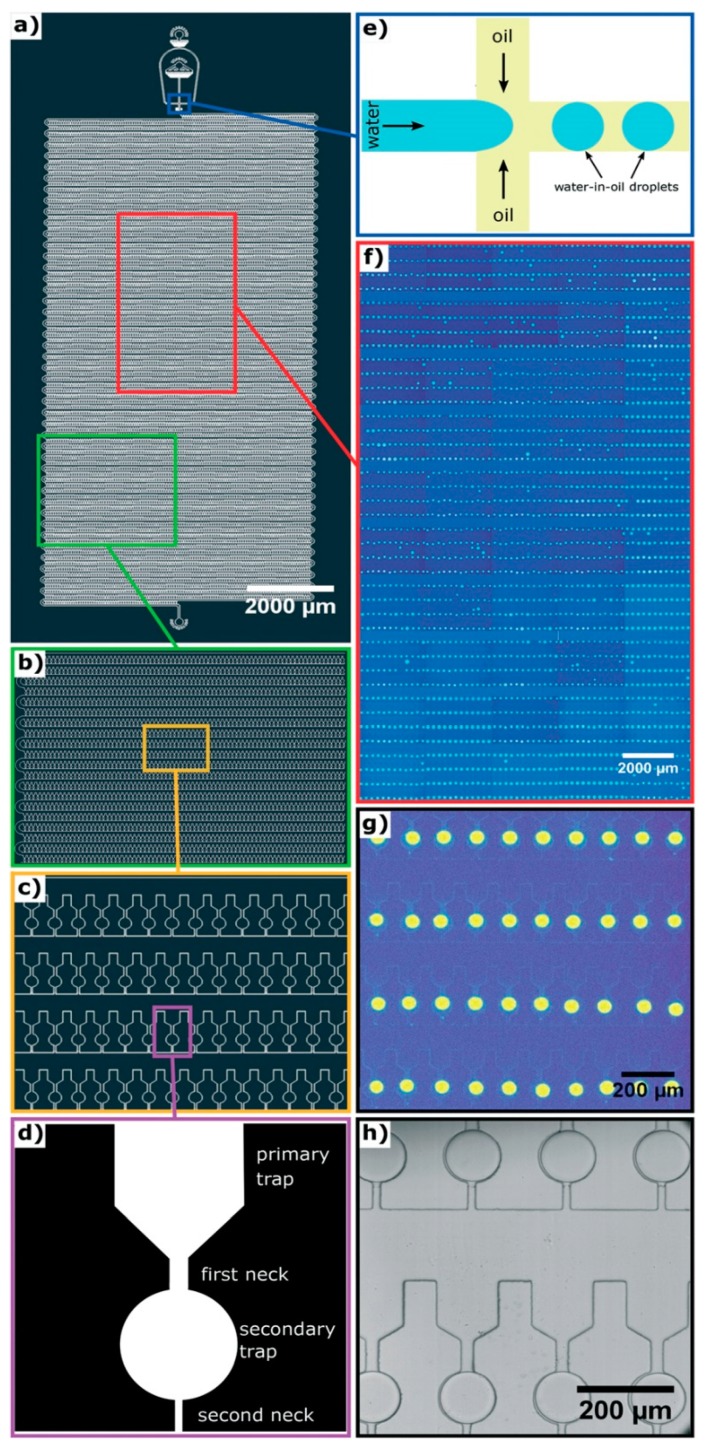
Design of a 10,000-microchamber microfluidic chip. (**a**) Details of the 10,000-microchamber microfluidic chip, (**b**,**c**) gradual expansion of a portion of the microchambers, (**d**) details of a microchamber, which comprise a funnel-like primary trap that opens into a spherical secondary trap via the first neck; the secondary trap opens into the main chamber via a secondary neck that is narrower compared to the first neck, (**e**) schematic of the junction at which water microdroplets are formed in oil via flow focusing, (**f**,**g**) gradual expansion of a portion of the microchambers with fluorescent microdroplets trapped within them, (**h**) bright field imaging of water microdroplets trapped within the microchambers. Republished with permission of Royal Society of Chemistry from: Observation of molecular self-assembly events in massively parallel microdroplet arrays. Toprakcioglu, Z. et al., Lab Chip, **2018** *18*, 3303. doi: 10.1039/c8lc00862k, copyright 2018; permission conveyed through Copyright Clearance Center, Inc.

In cases in which there may be many self-assembly pathways, e.g., amyloid assembly [48], it will be inefficient to perform the self-assembly process or vary the environmental conditions repeatedly to capture as many different self-assembly routes as possible. Instead, it will be much better to capture most, if not all, of the processes simultaneously with high throughput microfluidics as demonstrated by Toprakcioglu et al. [49]. In their work, a polydimethylsiloxane (PDMS) microchip with 10,000 microchambers was fabricated (Figure 3a–c). Within each microchamber, there are two traps. The primary trap leads into the secondary trap, which is spherical (Figure 3d). In order to trap water microdroplets within the microchambers, three parameters have to be adjusted precisely. Firstly, the channel (first neck, 25 μm in diameter) leading from the main chamber to the primary trap has to be thicker than the channel (second neck, 15 μm in diameter) that opens from the secondary trap to the main chamber. Secondly, the length ratio of the first neck and the second neck has to be more than 1.25. Last but not least, the surface tension of the microdroplet, which is determined by the interfacial surface tension between the water and oil being used, cannot be too high or too low. These three parameters permit the flow pressure to be adjusted so that it is high enough to push water through the first neck into the secondary trap, but not through the second neck back into the main chamber. This results in water droplets being trapped within the spherical secondary traps, with an efficiency of up to 99%. To test the capability of their chip, Toprakcioglu et al. studied the self-assembly of Phe-Phe by trapping subcritical concentrations of the peptide within the water droplets in the secondary traps. As the water slowly diffused through the PDMS chip, the concentration of Phe-Phe within the microdroplet increased gradually and eventually reached a critical concentration, triggering the self-assembly of Phe-Phe into nanotubes. Due to large number of microchambers available, i.e., 10,000 of them, it was possible to observe and monitor the stochastic nature of the self-assembly of Phe-Phe. The obvious strength of this set-up is that it can be easily adapted to study a wide variety of complicated processes.

**Figure 4:**
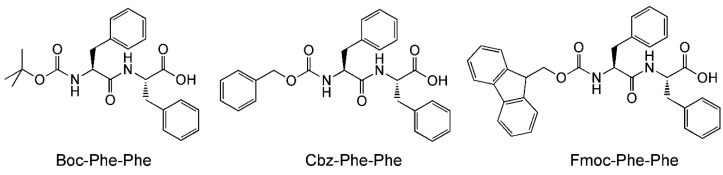
Molecular structures of Boc-Phe-Phe, Cbz-Phe-Phe, and Fmoc-Phe-Phe.

Another important aspect of peptide-mediated drug delivery is the interaction of the peptide nanostructures with the surfaces it comes into contact with. This is a vitally important aspect as the peptide nanocarrier can potentially come into contact with many different kinds of cell surfaces, surfaces with different properties (e.g., charges, cell-surface antigens). The properties of the peptide nanostructures have to be compatible to circumvent negative outcomes, e.g., loss of integrity of the nanostructure, loss of drug, lysis of healthy cells. With a microfluidics chip, Levin et al. have been able to visualize such an effect, albeit with a highly simplified system [50]. Levin et al. studied the effect of the nanostructures of three self-assembling peptides, i.e., *N*-tert-butoxylcarbonyl-phenylalanyl-phenylalanine (Boc-Phe-Phe), *N*-benzyloxycarbonyl-phenylalanyl-phenylalanine (Cbz-Phe-Phe), and *N*-(fluorenylmethoxycarbonyl)-phenylalanyl-phenylalanine (Fmoc-Phe-Phe) (Figure 4), to disrupt the water-oil interface. As the three peptides have limited solubilities in water, they were dissolved in a polar organic solvent first (ethanol for Boc-Phe-Phe and Cbz-Phe-Phe, dimethylsulfoxide for Fmoc-Phe-Phe). This microstream of peptide was then flow-focused into a microstream of water, which led to the dissolution of the peptides in water. In turn, the microstream of aqueous peptide was subjected to flow focusing by block copolymer surfactant/oil to generate surfactant-coated microdroplets of aqueous peptide in oil. Thus, by tweaking the initial concentration of peptide in the polar organic solvent, microdroplets with various peptide concentrations (subcritical, critical, supercritical) in oil could be obtained.

At a subcritical concentration of Boc-Phe-Phe, the peptide can be encapsulated within the aqueous microdroplet with no detectable self-assembly (by light microscopy) for up to 60 h. By allowing the microdroplets to evaporate in a controlled manner on a microscopic slide, the concentration of Boc-Phe-Phe was increased gradually beyond the critical aggregation concentration. At this point, the self-assembly of Boc-Phe-Phe into nanospheres could be observed to occur within 15 s. More importantly, it was observed that the nanospheres led to the disruption of the microdroplet/oil interface and were ejected in the surrounding oil at an initial rate of 22.5 ± 3.2 μm/s. This “jetting” phenomenon is indirectly dependent on the molecular structure of the self-assembling peptide, which impacts upon the nanostructures formed: while the rate of ejection of Fmoc-Phe-Phe was higher than Boc-Phe-Phe, that of Cbz-Phe-Phe was lower. On the other hand, polystyrene beads had no effect on the microdroplet/oil interface. The nature of the surfactant on the microdroplet in the oil also has an enormous effect on jetting: when Krytox, a perfluoropolyether-carboxylic acid, was the surfactant, jetting was not observed even at a high peptide concentration of 8 mg/mL.

Given that both the molecular properties of the peptide and the surfactant impact upon jetting, this indicates that there is physical interaction between the peptide nanostructures and the surfactant layer that separates the aqueous interior of the microdroplet and the oily surroundings. The physical interaction between the peptide nanostructure and the block copolymer surfactant is sufficiently strong and specific that the surface tension is decreased to the point surface breakage, leading to jetting. However, as the identity of the block copolymer surfactant is not reported, it is not possible to understand how the molecular differences among Fmoc-Phe-Phe, Boc-Phe-Phe, and Cbz-Phe-Phe lead to different interactions with the block copolymer surfactant or Krytox, which account for the differences in jetting rates of the peptide nanostructures. Nonetheless, these observations inform us of the necessity and possibility of tuning the physical properties of the peptide nanostructures so that they may be able to interact selectively with the membrane of the target cells and deliver the intended drug payload.

### 2.2. Utility of Microfluidics for Generating Self-Assembling Peptide-Based Microgels for Miniaturized Cell Cultures

All biomaterials need to be tested against appropriate cells to ascertain their cytocompatibility. This normally involves a cell culture, which would require substantial amounts of reagents such as cell culture medium and nutrients. In order to conserve excessive use of resources, it would be ideal to move towards miniaturized cell cultures for the testing of cytocompatibility, just as miniaturized bioreactors have been developed for therapeutics production [51]. By using only a few cells, the amount of resources required may be drastically reduced. This idea has been applied to the development of peptide microgel-based cell cultures [52]. Normally, the self-assembling peptide is dissolved in an appropriate solvent and allowed to gel over time. For peptide candidates that gel slowly over time, it is possible to utilize a microchannel to generate microgels within which cells may be cultivated. However, this would not be possible for a peptide candidate that gels very quickly.

**Figure 5:**
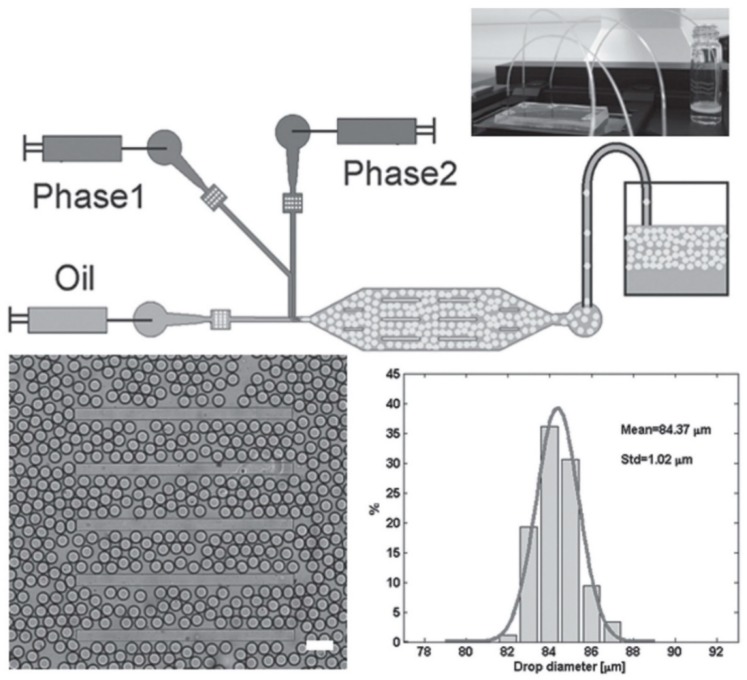
(Top half) Schematic of the microfluidic set-up that was used to mix the precursors of the self-assembling peptide along with an appropriate enzyme. After mixing, the self-assembling peptide was produced, which gelled in situ to produce the microgels. (Bottom half) Illustration of the uniformity of the generated microgels. The mean width of the microgels was 84 μm, with a standard deviation of only 1 μm, demonstrating the ability of this microfluidic technique to generate uniformly sized microgels. Republished with permission of John Wiley and Sons Inc, from: Biocatalytic self-assembly of nanostructured peptide microparticles using droplet microfluidics, Bai, S. et al., Small **2014** 10, 285. doi: 10.1002/smll.201301333, copyright 2014; permission conveyed through Copyright Clearance Center, Inc.

To overcome this problem, Bai et al. have designed a microfluidic set-up in which two differentially protected amino acids could be separately co-injected into two different continuous aqueous streams that are focused into a stream of fluorous oil containing 2% w/w of polyethyleneglycol-perfluoropolyether (PEG-PFPE) [53]. As the two streams are laminar, the contents in the respective streams do not mix until they collide and form microdroplets in the presence of the oil at the T-junction (Figure 5, top half). PEG-PFPE acts as a surfactant that stabilizes the water microdroplets: presumably, the PEG component faces the water interior whereas the PFPE component faces the fluorous oil exterior. One set of optimized flow conditions, coupled with the laminar partitioning within the elongated octagonal chamber, led to the production of PEG-PFPE-stabilized microdroplets with a mean diameter of 84 μm and narrow size distribution (standard deviation = 1 μm) (Figure 5, bottom half).

**Figure 6:**
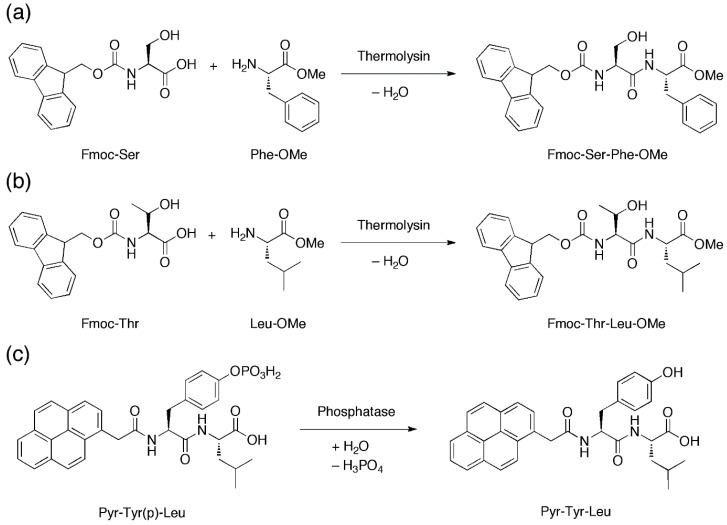
(**a**,**b**) Amide coupling reactions that are mediated by thermolysin to produce Fmoc-Ser-Phe-OMe and Fmoc-Thr-Leu-OMe. (**c**) Dephosphorylation that is mediated by phosphatase to produce Pyr-Tyr-Leu. Upon production, these self-assembling peptides can gel in situ to generate microgels.

With their microfluidic set-up, Bai et al., introduced two peptide precursors, e.g., *N*-(fluorenylmethoxycarbonyl)-serine (Fmoc-Ser) and phenylalanine-*O*-methylester (Phe-OMe), via the two separate aqueous streams. In one of the two streams the enzyme thermolysin was also present. Thermolysin catalyzes the amide coupling of Fmoc-Ser and Phe-OMe to furnish Fmoc-Ser-Phe-OMe (Figure 6a) in the microdroplets when the laminar aqueous streams are flow-focused into microdroplets in the presence of the fluorous oil. This allows the concentration of Fmoc-Ser-Phe-OMe, a known hydrogelating peptide [54], to gradually build up until the critical hydrogelating concentration is reached, leading to hydrogelation of the microdroplets to form microgels. The generality of this approach can be gleaned by the thermolysin-catalysed amide coupling of *N*-(fluorenylmethoxycarbonyl)-threonine/leucine-*O*-methylester (Fmoc-Thr/Leu-OMe) and the phosphatase-catalyzed dephosphorylation of 2-pyrenylacetylphosphotyrosine-leucine (Pyr-Tyr(p)-Leu) to generate respectively Fmoc-Thr-Leu-OMe [55] (Figure 6b) and 2-pyrenylacetylphosphotyrosine-leucine (Pyr-Tyr-Leu) [56] (Figure 6c) microgels.

**Figure 7:**
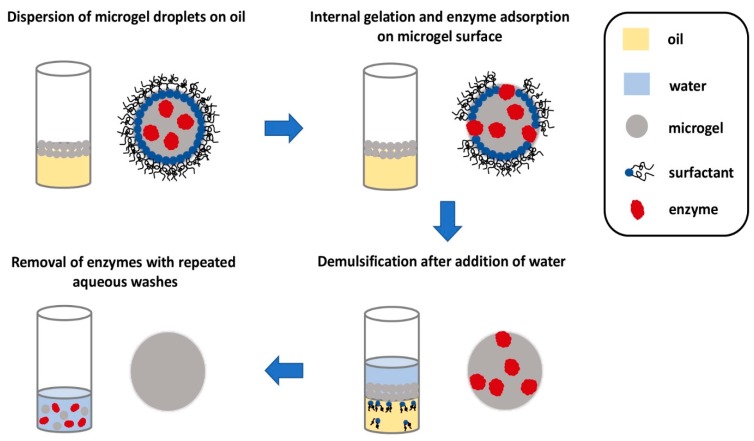
Pictorial representation of the recovery of homogeneous microgels. As the microgel droplets are less dense, they float to the surface to form an emulsion. The presence of the enzymes effected demulsification. The microgel droplets can then be removed and washed repeatedly with water to remove the enzyme to furnish pure microgel droplets. Redrawn from: Biocatalytic self-assembly of nanostructured peptide microparticles using droplet microfluidics, Bai, S. et al., Small **2014** 10, 285. doi: 10.1002/smll.201301333.

After the microgels are enzymatically generated, a foamy emulsion is formed on top of the denser fluorous oil. It is important to be able to separate the microgels from the emulsion in a facile manner before they can be utilized in any applications. Fortuitously, the enzymes facilitate demulsification and subsequent recovery of the microgels. After the microgel forms, the enzymes seemingly translocate to the surface of the microgels, as suggested by fluorescence microscopy images. The presence of enzymes on the surface of the microgel permits demulsification to happen after an aqueous buffer is added to the emulsion. Subsequently, the microgels in the aqueous layer can be removed and washed with more water to remove the surface-bound enzymes, furnishing microgels almost free of the enzymes (Figure 7). This final step is important as the enzymes might interfere with the cell culture or any other applications that these microgels are to be used for. Scanning electron microscopy indicated that the size of the microgels remained essentially unchanged after this procedure. This microfluidic set-up is evidently elegant, allowing quick gelling peptide microgels to be formed and isolated for miniaturized cell culture.

**Figure 8:**
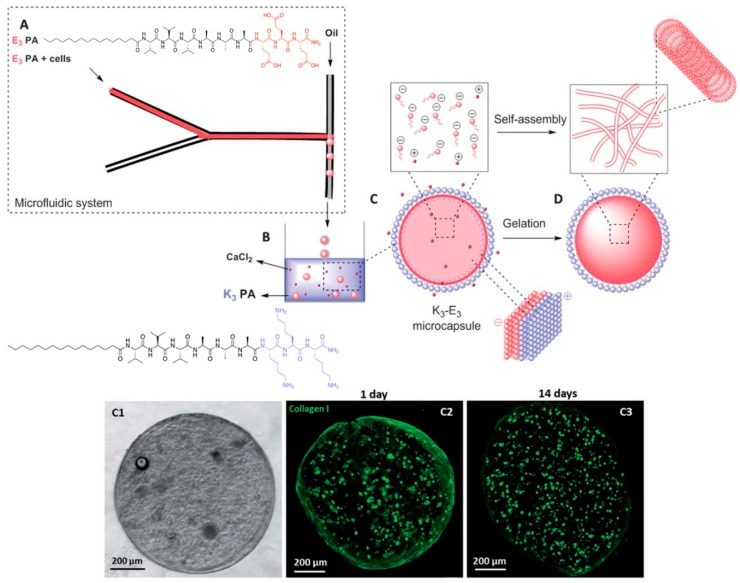
(Top half) Stepwise formation of charge-paired peptide nanoparticles. An aqueous stream of C_16_-(valine)_3_-(alanine)_3_-(glutamic acid)_3_ (E_3_-PA) is flow-focused into a stream of oil to generate uniformly-sized microdroplets. On exposure to an aqueous pool of C_16_-(valine)_3_-(alanine)_3_-(lysine)_3_ (K_3_-PA), the mineral oil floats away and K_3_-PA co-assembles with E_3_-PA on the microdroplet surface. Diffusion of CaCl_2_ into the microgel core leads to self-assembly and consequent hydrogelation of E_3_-PA. (Bottom half) Illustration of the encapsulation of fibroblasts within a microcapsule. (C1) Phase contrast microscopic image. (C2) Confocal fluorescence microscopic image of collagen I produced by fibroblasts after 1 day, (C3) after 14 days. C2 and C3 show that there is an increase in the amount of collagen I in the microcapsule after 14 days, indicating that the fibroblasts are actively modifying their microenvironment. Republished with permission of Royal Society of Chemistry, from: Peptide-based microcapsules obtained by self-assembly and microfluidics as controlled environments for cell culture, Ferreira, D. S. et al., Soft Matter **2013**, 9, 9237. doi: 10.1039/c3sm51189h, copyright 2013; permission conveyed through Copyright Clearance Center, Inc.

Another peptide hydrogel system that can greatly benefit from microfluidics is a two-peptide system in which both peptides are oppositely charged. Normally, the mixing of oppositely charged species will lead to indiscriminate electrostatic coupling to produce a mixture of charge-paired clusters of various orders (e.g., 10 or 22 charge-pair clusters). This would not be conducive towards generating uniformly-sized peptide microgel cell cultures. To overcome this problem, Ferreira et al. utilized microfluidics to prepare a sample of negatively charged microgel capsules that are coated with a layer of positively charged peptide (Figure 8) [57]. To achieve this, they focused a solution of C_16_-(valine)_3_-(alanine)_3_-(glutamic acid)_3_ (E_3_-PA), negatively charged peptide amphiphile, into a stream of mineral oil. Due to the immiscibility of the aqueous solution with the mineral oil, a steady stream of aqueous microdroplets of negatively charged peptides is formed. This stream is subsequently extruded into an aqueous pool of C_16_-(valine)_3_-(alanine)_3_-(lysine)_3_ (K_3_-PA), a positively charged peptide amphiphile. As the mineral oil surrounding the microdroplets slowly peel away due to its lower density than water, the negatively charged E_3_-PA microdroplets are gradually exposed to the positively charged K_3_-PA. Co-assembly between E_3_-PA and K_3_-PA on the surface of the microdroplet leads to a neutral shell of E_3_-PA/K_3_-PA. Last but not least, the addition of CaCl_2_ can lead to the hydrogelation of the microdroplet core to form the microgel: diffusion of CaCl_2_ into the liquid core of the microdroplets induces the self-assembly of the negatively-charged peptide amphiphile and subsequent hydrogelation [58]. This allows the creation of stable bifunctional microgels that certainly could not be achieved simply by mixing the positively and negatively charged peptides together. This approach also has the advantage of producing well defined multilayered microgels, compared to the homogenous microgel system of Bai et al.

The stability of these microgels is evident from the lack of swelling when they are immersed in either water or phosphate-buffered saline. The retention of the physical integrity of the microgels after mechanical agitation by glass beads shows that the microgels are also physically robust. These observations indicate that these microcapsules are extremely amenable to physical handling, which lends itself well to the regular changes of the medium as required during cell culture. These microgels were also demonstrated to be permeable to dextran as large as 155 kDa, with the rate of permeation adjustable with different thickness of the E_3_-PA/K_3_-PA shell, which in turn can be controlled by the concentration of K_3_-PA that the E_3_-PA-microdroplets are exposed to. More importantly, the permeability of the microgels meant that it is indeed feasible to culture cells within the microgel core since low molecular weight nutrient/waste product exchange is possible.

The potential of these microgels as miniaturized cell cultures was amply demonstrated by the successful culture of human dermal fibroblasts within the microgels. The live/dead assay indicated that the encapsulated cells were viable for up to 14 days. The amount of DNA within the microgel almost doubled within the same duration, implying that cells also proliferated. In addition, the cells retained their fibroblastic characteristic of modifying their environment via the production of collagen I: confocal fluorescence microscopy revealed the presence of collagen I, and the increase in its quantity, over the culture duration. The observation of an external fibrillar network distinct to that of self-assembled E_3_-PA by scanning electron microscopy also supports the presence of collagen I. E_3_-PA concentration also affected the extent of collagen I synthesis by the fibroblasts: with 0.5% w/w E_3_-PA, there was a lot more collagen I than with 1% or 2% w/w E_3_-PA within the microgel. All these observations indicate that the microgels provide a hospitable environment for the cells to survive, proliferate, and actively engage the microenvironment. Last but not least, it is even possible to co-culture a second type of cell, i.e., keratinocytes, on the surface of the microgels, within which fibroblasts are cultivated. This opens up the possibility to utilize microfluidics to create 3D microenvironments in which different cell types are spatially well defined within a microgel capsule for the study of cell-cell interaction that occurs in a tissue. It would certainly also be possible to study cell-cell interaction between microgel capsules that have different cell types attached to their surfaces. More importantly, this would be a relatively simple way to tune the viscoelasticity of the microgel capsules, which has been known to critically affect the differentiation outcome of stem cells [59].

**Figure 9:**
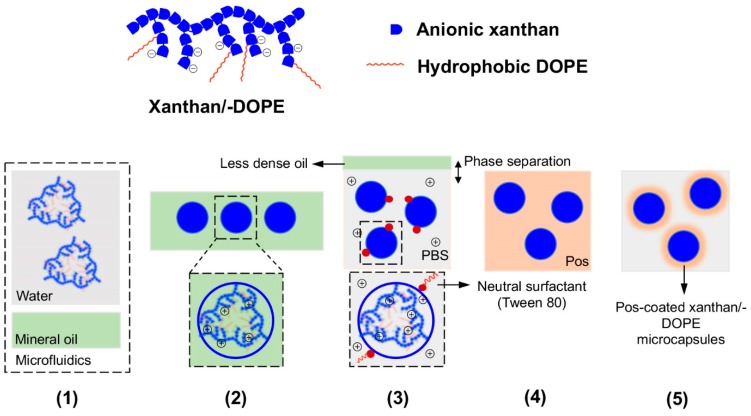
(Top half) Cartoon of the structures of the negatively-charged xanthan (blue blocks only) and xanthan-DOPE (blue blocks and red lines). (Bottom half) Pictorial representation of the production and isolation of coated microgels from the microfluidic set-up; Pos refers to the positively-charged Lys_2_-(Gln-Leu)_6_-Lys_2_ or polylysine. (1) Xanthan or xanthan-DOPE (xanthan/-DOPE) is dissolved in the water microstream and focused into mineral oil in the microfluidic set-up, (2) uniformly sized microdroplets of xanthan/-DOPE formed in the mineral oil after flow focusing, (3) exposure of the microdroplets to phosphate-buffered saline (PBS) led to the phase separation of the microdroplets from the mineral oil and stabilization of the microdroplet surface by Tween 80, (4) exposure of the microdroplets to Pos led to electrostatic attraction between xanthan/-DOPE and Pos, and consequently peptide self-assembly on the microdroplet surface, and (5) formation of stable Pos-coated xanthan/-DOPE microcapsules, with the thickness of the coat dependent on Pos concentration in step 4. Redrawn from: Fabrication of phospholipid-xanthan microcapsules by combining microfluidics with self-assembly, Mendes, A. C. et al., Acta Biomater. **2013** 10, 285. doi: 10.1016/j.actbio.2013.01.035.

Besides peptidic hydrogels, it is also possible to generate multilayered microgels from different types of substrates. Mendes et al. have employed microfluidics to prepare a xanthan (a negatively charged polysaccharide) microgel that is coated with a self-assembling positively charged peptide (Figure 9) [60]. They injected an aqueous solution of xanthan slowly into mineral oil, which led to the formation of xanthan microdroplets within the microfluidic device. On releasing the xanthan microdroplets into a reservoir of lysine_2_-(glutamine-leucine)_6_-lysine_2_ (Lys_2_-(Gln-Leu)_6_-Lys_2_), the surface of the xanthan microdroplet is coated with the self-assembling peptide. The thickness of the peptide coat can be varied by the Lys_2_-(Gln-Leu)_6_-Lys_2_ concentration that the xanthan microdroplets are exposed to: with 0.1% and 0.5% w/w of peptide, the thickness of the peptide coat differs by one-fold (5–10 μm). The thickness of the peptide coat confers different degrees of rigidity to the microcapsules, with a thicker coat imparting greater resistance to the microcapsules to breakage during physical agitation with glass beads. The thickness of the peptide coat also has implications on the permeability of the microcapsules to molecules. As expected, the thicker the peptide coat, the less permeable the microcapsules were to the immunoglobulin G antibody (M_w_ = 146–155 kDa) they were exposed to [61]. The peptide coat, however, is certainly readily permeable to small molecules, as suggested by the possibility of culturing ATDC5 chondrocyte cells within the microcapsule. Over 21 days, AlamarBlue assay indicated that the encapsulated chondrocytes were metabolically active, live/dead assay indicated that the cells were alive, and DNA quantification assay indicated that the cells were proliferating. This meant that exchange of nutrients/waste products occurs readily between the microcapsule interior with the external medium, which is a prerequisite for the survival of the cells within the microcapsules.

Similarly, xanthan-DOPE (1,2-**d**i**o**leoyl-sn-glycero-3-**p**hospho**e**thanolamine)/polylysine microcapsules have also been prepared using the same method (Figure 9) [62]. In this set-up, xanthan-DOPE serves as the anionic component that forms the core of the microcapsule whereas polylysine serves as the cationic component that coats the surface of the microcapsule. Xanthan-DOPE was dissolved in HEPES buffer, after which it was injected into mineral oil in a microfluidic device. This led to the production of microcapsules of around 465 ± 55 μm in diameter. On exposure to phosphate-buffered saline, the NaCl and KCl present may help to solidify the wall of the microcapsules. Interestingly, the presence of polylysine constricts the microgel capsules to about 283 ± 13 μm in diameter. As with xanthan/Lys_2_-(Gln-Leu)_6_-Lys_2_ microcapsules, the xanthan-DOPE/polylysine microcapsules can fully support the metabolic activity, survival, and proliferation of ATDC5 chondrocyte cells. However, since the core of the xanthan-DOPE/polylysine microcapsules is somewhat hydrophobic, it might even be possible to encapsulate hydrophobic drugs and adapt the microcapsules for drug delivery [63].

## 3. Conclusions and Outlook

The current focus review illustrates the operational simplicity of using microfluidics to study peptide self-assembly, peptide-interface interaction, and generate uniformly-sized self-assembling peptide-based microgels for miniaturized cell cultures. Despite the impressive advances already made, there are two technical areas that require further attention. Firstly, many of these systems utilize fluorous oils as one of two complementary phases in the generation of the microcapsules. While the phase separation of fluorous oils with aqueous solutions is excellent and they do not corrode the polymeric materials used to construct the microfluidic chips, fluorous oils are expensive. To bring down the cost of operations, it would be ideal to switch to more common organic liquids. In this case, however, the material of the microfluidic chip would have to be modified to be able to resist organic liquids and yet be amenable to the fabrication of the microchannels of the microfluidic chip. Secondly, it would be important to adapt existing spectroscopic tools to be able to yield more information on the mentioned processes. Indeed, efforts to integrate more optical spectroscopic techniques with microfluidics are already underway [64]. These include absorbance and fluorescence spectroscopy [65], terahertz time-domain spectroscopy [66], and optical tweezer technology [67]. However, more techniques, e.g., Raman spectroscopy, circular dichroism spectroscopy, and nuclear magnetic resonance (NMR) spectroscopy, that can furnish complementary information are required, in particular for monitoring a large number of events simultaneously as in the case of Toprakcioglu et al. [49]. However, these techniques (especially NMR spectroscopy) will require further technological developments in assimilating these techniques with the microfluidic chip. Combined with the emerging ability to computationally design self-assembling peptides [68,69,70,71], it will become easier and faster to design niche nanoparticles and microgels with desirable properties for different cell types. It will certainly not be long before precision peptide nanoparticles and microgels can be prepared to fulfill their nanobiotechnological potential.
